# Exploring the Anticancer Potential of Lamivudine-Loaded Polymeric Nanoparticles: *In Vitro* Cytotoxicity, Tissue Deposition, Biochemical Impact *In Vivo*, and Molecular Simulations Analysis

**DOI:** 10.1021/acsabm.5c00182

**Published:** 2025-05-19

**Authors:** Natália Cristina Gomes-da-Silva, Alicia de Faria Almeida, Patrícia Severino, Mohammed Al-Qahtani, Luciana Magalhães Rebelo Alencar, Pierre Basílio de Almeida Fechine, Eduardo Ricci-Junior, Laura Fernanda Osmari Vendrame, João Augusto Pereira da Rocha, Solange Binotto Fagan, Ralph Santos-Oliveira

**Affiliations:** † Brazilian Nuclear Energy Commission, Nuclear Engineering Institute, Laboratory of Nanoradiopharmacy and Synthesis of New Radiopharmaceuticals, Rio de Janeiro 21941906, RJ Brazil; ‡ Cyclotron and Radiopharmaceuticals Department, King Faisal Specialist Hospital and Research Center (KFSHRC), Riyadh 11564, Saudi Arabia; § 37892 Biophysics and Nanosystems Laboratory, Federal University of Maranhão, Department of Physics, São Luis 65065690, MA Brazil; ∥ Grupo de Química de Materiais Avançados (GQMat), Departamento de Química Analítica e Físico-Química, 28121 Universidade Federal do Ceará-UFC, Campus do Pici, CP 12100, Fortaleza, 60451-970, CE Brazil; ⊥ Federal University of Rio de Janeiro, School of Pharmacy, Rio de Janeiro 21941900, RJ Brazil; # Franciscan University, Rua dos Andradas 1614, Santa Maria 97010-100, RS Brazil; 7 Laboratory of Modeling and Computational Chemistry, Federal Institute of Education, Science and Technology of Pará (IFPA), Campus Bragança 68600-000, PA Brazil; 8 Rio de Janeiro State University, Laboratory of Radiopharmacy and Nanoradiopharmaceuticals, Rio de Janeiro 23070200, RJ Brazil

**Keywords:** oncology, nanotechnology, treatment, therapy, radiolabeling

## Abstract

Lamivudine is a synthetic nucleoside analogue to cytosine with a modified sugar moiety. It has potent action against Human Immunodeficiency Virus and chronic hepatitis. Recently, studies have also shown that lamivudine (3TC) can induce apoptosis in cancer cells and inhibit their proliferation, including breast cancer. We prepared polymeric nanoparticles using the double emulsification technique to incorporate polycaprolactone (PCL) as the polymer and lamivudine as the active compound. The nanoparticles were characterized by atomic force microscopy and dynamic light scattering. Then we carried out a full set of *in vitro* and *in vivo* analyses, including measurement of cytotoxicity, radiolabeling, biodistribution and biochemistry. The results showed the formation of 273 nm spherical nanoparticles with monodisperse behavior (PDI = 0.052). The radiolabeling with ^99m^Tc demonstrated the feasibility of the direct radiolabeling process. The cytotoxicity corroborated the potential against the triple-negative breast cancer line (MDA-MB-231). The biodistribution assay revealed high uptake in the liver, small and large intestines and bladder, besides the presence of nanoparticles in the urine. The *in vivo* biochemistry analysis showed alterations in some enzyme levels, including: alanine aminotransferase (ALT), aspartate aminotransferase (AST), gamma GT (GGT), creatinine (CRE), amylase (MAS), lactate dehydrogenase pyruvate (LDH-P) and glucose (GLU). Finally, we performed theoretical studies of molecular docking, molecular dynamics and interactions between lamivudine and key proteins regulating necroptosis, including epidermal growth factor receptor (EGFR), receptor-interacting protein kinase 1 (RIPK1), and receptor-interacting protein kinase 3 (RIPK3). Theoretical results showed lamivudine’s adaptability to the binding sites of these proteins, with potential for optimization to enhance hydrophobic interactions and binding affinity. The findings demonstrated the efficacy of lamivudine against breast cancer cells, and the need to better understand the interplay of nanosystems with biochemical parameters.

## Graphical abstract

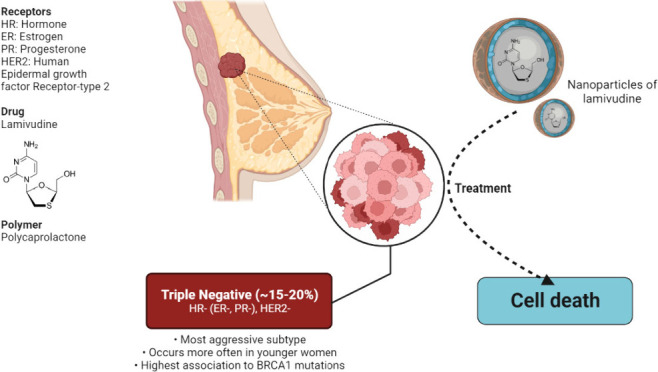

## Introduction

Breast cancer is the most frequently diagnosed cancer among women worldwide, accounting for approximately 25% of all cancer cases. While predominantly affecting women, men are also susceptible, albeit at significantly lower rates.^1–3^ Globally, breast cancer represents about 11.7% of all new cancer cases annually, with approximately 2.3 million cases diagnosed each year.^1,4^ Globally, breast cancer represented about 11.7% of all new cancer cases annually, with approximately 2.3 million diagnoses in 2022.^1,4^ It remains a leading cause of cancer-related mortality, responsible for an estimated 670,000 deaths in 2022. Projections indicate that by 2050, new cases and deaths will have increased by 38% and 68%, respectively, disproportionately affecting countries with a low Human Development Index (HDI). Disparities in survival rates between developed and developing countries underscore the importance of accessible healthcare and sound public health policies.

Drug repurposing, or repositioning, is a strategic approach to identify new therapeutic uses for existing drugs, offering a faster and more cost-effective alternative to traditional drug development.^5,6^ Lamivudine, a nucleoside reverse transcriptase inhibitor (NRTI) prescribed to treat Human Immunodeficiency Virus (HIV) and hepatitis B, has shown potential in oncology, by inducing necroptosis and DNA damage, stimulating interferon gene (STING) pathway activation, and inhibiting mechanisms that can help overcome cancer resistance to conventional therapies.^7–13^

Necroptosis, a distinct programmed cell death pathway, differs from apoptosis by causing cell membrane rupture and the release of intracellular components. This process can enhance antitumor immunity by activating immune responses through damage-associated molecular patterns (DAMPs).^14–16^ Lamivudine’s ability to induce necroptosis enables a novel therapeutic strategy, particularly for treatment-resistant cancers.^17,18^ Additionally, lamivudine disrupts DNA repair mechanisms, increasing cancer cell sensitivity to genotoxic stress, hence leading to apoptosis or senescence.^19–21^

The exploration of lamivudine’s impact on the STING pathway is another promising avenue in cancer immunotherapy. The STING pathway detects cytosolic DNA from cellular stress or damage, triggering an immune response via type I interferons and cytokines.^22–24^ By activating this pathway, lamivudine may enhance the immune system’s ability to eliminate cancerous cells.^25,26^

Lamivudine also appears to interfere with telomerase reverse transcriptase (TERT), a key component in cellular aging and oncogenesis. TERT overexpression in many cancers helps cells evade normal proliferative limits. While the precise mechanism by which lamivudine affects TERT remains under investigation, evidence so far suggests it might limit telomere maintenance, thus reducing cancer cell survival and promoting senescence or apoptosis.^27–31^

Combining drug repurposing with nanotechnology improves therapeutic efficacy. Nanoparticles of lamivudine (LamNPs) enhance bioavailability, stability and target-specific drug delivery. Various methods, including solvent evaporation, coacervation, emulsion-diffusion and nanoprecipitation, have been employed to produce LamNPs, which demonstrate superior pharmacokinetics and therapeutic performance in animal models compared to free drugs.^32–35^ Studies suggest that LamNPs have dose-dependent cytotoxic effects and high anticancer activity, particularly in breast and lung cancer models.^36–38^

Targeting necroptosis-related proteins such as receptor-interactingInteracting protein kinase (RIPK1), receptor-interacting protein kinase 3 (RIPK3) and epidermal growth factor receptor (EGFR) through nanoencapsulation may further optimize treatment outcomes. Recent studies indicate that LamNPs enhance drug stability and affinity at binding sites, improving specificity and reducing off-target effects. This novel therapeutic approach of combining drug repurposing and nanotechnology is a promising strategy for overcoming cancer resistance and improving patient outcomes.^39^

We prepared and characterized polymeric LamNPs and evaluated their potential as a breast cancer nanodrug both *in vitro* and *in vivo*. Additionally, we performed molecular docking and molecular dynamics (MD) studies of EGFR, RIPK1 and RIPK3, key proteins in necroptosis, to investigate lamivudine’s ability to induce cancer cell death.

## Materials and Methods

### Reagents

All reagents and solvents used in this study were purchased from Sigma-Aldrich (Brazil).

### Polymeric Nanoparticle Production

PCL nanoparticles were prepared by the double emulsion solvent evaporation technique as described by Ekinci et al. (2022). Briefly, 2 mL of 1% poly­(vinyl alcohol) (PVA) solution with lamivudine was dripped into 4 mL of dichloromethane and PCL (2-Oxepanone homopolymer, 6-Caprolactone polymer, *M*_n_ 80,000). The drug/polymer ratio in all cases was 1:1. The mixtures were sonicated (UP100H, Hielsher) for 4 min at 100% amplitude to produce a water-in-oil (W/O) emulsion. This emulsion was emulsified again with 8 mL of PVA 1% (w/v) and processed for 4 min to produce a W/O/W emulsion. The solvents were evaporated under reduced pressure for 2 h at 25 °C (IKA HB eco rotary evaporator). The nanoparticles were recovered by centrifugation (20,000 rpm for 20 min) (Optima XE-90 Ultracentrifuge, Beckman Coulter) and washed twice with distilled water to remove the excess PVA.

### Size Determination by DLS

Particle size and size distribution, mean size and polydispersity index (PDI) were determined by dynamic light scattering (DLS) using a Nano ZS Zetasizer (Malvern Instruments, UK). Measurements were performed in triplicate at 25 °C, and the laser incidence angle with the sample was 173° using a 12 mm^2^ quartz cuvette. The mean ± standard deviation (SD) was calculated.

### Atomic Force Microscopy (AFM)

#### Sample Preparation

The particle solutions were diluted to approximately 10^9^ to 10^10^ particles per 1 cm^3^. The solutions were added dropwise in freshly cleaved mica and left to dry in a vacuum chamber protected from contamination.

#### AFM Setup

Atomic force microscopy (AFM) experiments were performed with a Multimode 8 microscope (Bruker, Santa Barbara, USA) using the Nanoscope software (Bruker) in PeakForce Quantitative Nanomechanics (QNM) mode.^15^ The experiments were performed with a cantilever spring constant of 0.4 N/m and a nominal tip radius of 2 nm. The experiments were also performed with a scan resolution of 256 × 256 lines and a scan frequency of 0.5 Hz. The images were analyzed using the Gwyddion 2.60 and Nanoscope Analysis 2 programs. For the particle diameter analysis, AFM maps of 10 μm containing hundreds of nanoparticles were used in Nanoscope particle analysis mode, and the mean diameter of the lamivudine nanoparticles was calculated.

### UV–Vis Spectrophotometry

#### Encapsulation Efficiency

To evaluate the encapsulation efficiency (EE%) by UV–vis spectroscopy, we used an indirect method to analyze the supernatant and nanoparticle samples. After synthesizing the lamivudine nanoparticles, the structures were ultracentrifuged and the supernatant was observed, and its absorbance were measured at a wavelength of 271 nm in a spectrophotometer (Kasvi, K37-UVVIS). The spectra were registered in the range of 190–1100 nm using a 1 cm silica cuvette at room temperature. The value, in percent, was obtained by the following formula [Disp-formula eq1].

1 $$EE\%=\frac{Encapsulatedmassofdrug}{Totalmassofdrugsynthesized}\times 100$$

#### Release Profile

The drug release profile was analyzed using the dialysis bag method. Initially, the dialysis bag was immersed in distilled water to ensure proper hydration of the membrane. Subsequently, 1 mL of lamivudine nanoparticles at a concentration of 44.8 mg/mL was introduced into the hydrated dialysis bag. This bag containing the lamivudine nanoparticles was then placed in a beaker filled with 0.2 L of distilled water. The beaker was maintained at a constant temperature of 37 °C and stirred at a rate of 100 rpm, utilizing an IKA C-MAG HS 7 magnetic stirrer equipped with heating capability. Sampling for analysis occurred at predetermined intervals from 0 to 30 h. At each time point, 4 mL of the solution was extracted from the beaker in triplicate for UV–vis spectroscopy (Kasvi, K37-UVVIS) analysis. It is important to note that each time a sample was removed for analysis, an equivalent volume of distilled water was replenished to maintain a consistent volume throughout the experiment.

#### Cell Culture

MDA-MB-231 human breast cancer cell lines were obtained from the American Type Culture Collection (ATCC HTB-26) and maintained in a high-glucose medium (Dulbecco’s Modified Eagle Medium - DMEM) supplemented with 10% (v/v) fetal bovine serum, 2 mM L -glutamine and 1% (v/v) penicillin/streptomycin. The cells were grown and expanded in T75 cm^2^ flasks and kept in a humidified oven with 5% CO_2_ and 37 °C. Monolayer assays were performed in 24-well sticky plates

#### Cell Viability

For cell viability, 1 × 10^4^ cells were cultured in 96-well plates. After adherence for 24 h, they were treated with 10, 50, or 100 μg/mL of LamNPs or PCL nanoparticles (100 μg/mL) and evaluated after 24 h. At the end of the designated time, a MTT solution (Sigma) was added at 1 mg/mL in the culture and incubated for 2 h. After the incubation, formazan crystals were dissolved with DMSO, and absorbance was measured with a multiplate spectrophotometer at a wavelength of 450 nm.

### *In Vivo* Biodistribution: Tissue Deposition

#### Labeling Process with ^99m^Tc

The technetium-99m labeling process was initially performed by injecting 0.3 mL of stannous chloride (SnCl^2^) (80 μL/mL) (Sigma-Aldrich) with 1 mL of [^99m^Tc] at 1.99 mCi and incubating the mixture for 10 min. Next, 0.6 mL of NP solution was added to the solution and incubated for another 10 min to mark its structures.

#### Quality Control of the Labeling Process with Tc-99m

In order to confirm the efficacy of the labeling process, radio thin-layer chromatography (RTLC) was applied using Whatman #1 filter paper using 2 l of the ^99m^Tc-LamNPs and acetone (Sigma-Aldrich) as mobile phase at times of 0, 1, 2, 3, and 24 h. The radioactivity of the strips was verified in a γ-counter (Hidex, Turku, Finland). The RTLC was performed in triplicate for each time interval.

#### Animals

Animal experiments were performed with C57bl/6 males (n = 3), weighting between 25 and 30 g (3–5 weeks old). Animals were housed one per cage under controlled conditions of luminosity (12:12 h light/dark cycle) and temperature (21.0 ± 1.0 °C), with free access to water and standard chow. All procedures were approved by the State University of Rio de Janeiro Animal Care and Use Committee (Rio de Janeiro, RJ, Brazil; protocol number CEUA/8059100220/2021), which is consistent with the United States National Institute of Health Guide for Care and Use of Laboratory Animals (National Research Council, 1996).

#### Animal Preparation

Animals were anesthetized without prior fasting by intraperitoneal injection of ketamine (100 mg/kg) and xylazine (20 mg/kg).

#### Design Protocol

For the biodistribution/tissue deposition studies, 90 μCi (3,3 MBq)/0.1 mL of ^99m^Tc-LamNPs (nanoparticlesnanoparticle radiolabeled with technesium-99m) was injected intraperitonially (i.p.) to evaluate the systemic behavior in healthy animals. Animals were sacrificed 4 h after injection by using an excess of anesthesia (isoflurane chamber). The blood and organs of interest (heart, brain, stomach, intestine, bladder, kidneys, lungs, liver and spleen) were immediately dissected and weighed for quantitative estimation of gamma counts using a gamma counter (Hidex, Turku, Finland). Results were expressed as percentage of injected dose per gram of organ (%ID/g).

#### Biochemistry Analysis

Blood samples were collected by cardiac puncture from healthy mice treated (intervention group) with LamNPs 24 h after intraperitoneal administration (n = 3 per group). Then the blood samples (0.5 mL) were added into microtubes containing 0.5 mL of the anticoagulant heparin (Sigma-Aldrich, Brazil). Plasma was separated by centrifugation (5000 rpm, 5 min, 4 °C) and the samples were processed according to the manufacturer’s instructions (Bioclin, MG, Brazil) to determine the enzymatic activities of alanine aminotransferase (ALT), aspartate aminotransferase (AST), gamma GT (GGT), creatinine (CRE), lactate dehydrogenase pyruvate (LDH-P), glucose (GLU) and amylase (MAS).

#### Statistical Analyses

For the *in vitro* experiments, values are expressed as mean ± standard deviation (SD). Differences between groups were tested for significance by one-way ANOVA followed by the Tukey multiple comparison test using the GraphPad Prism 8.1 software (GraphPad Software, San Diego, CA, USA). A *p* value of ≤ 0.05 was considered significant.

#### Molecular Docking

To prepare the ligands used in molecular docking studies, the structures of lamivudine were taken from PubChem (CID: 60825). The crystallographic structures of the ligands of the EGFR, RIPK1, and RIPK3 proteins were used according to their respective PDB files (1M17, 4NEU and 7MX3). All molecules were prepared with definition of the protonation states and calculation of partial charges using the RESP model^40^ via Gaussian 16.^41^ The protonation states of the ligands were determined considering a pH of 7.4, using the Open Babel software^42^ to predict the predominant species at this physiological pH. The H++ server^43^ was used to add hydrogens and adjust the protonation states of the proteins to the mentioned physiological pH values. The molecular docking simulations were performed with the GOLD program version 2024.2. To validate the protocol, the crystallographic ligands associated with the proteins (AQ4 ([6,7-bis­(2-methoxy–ethoxy)­quinazoline-4-yl]-(3-ethynylphenyl)­amine) for EGFR, Q1A (1-[4-(1-aminoisoquinolin-5-yl)­phenyl]-3-(5-*tert*-butyl-1,2-oxazol-3-yl)­urea) for RIPK1, and ZOV (3-(1,3-benzothiazol-5-yl)-7-(1,3-dimethyl-1H-pyrazol-5-yl)­thieno­[3,2-*c*]­pyridin-4-amine) for RIPK3) were redocked. The root-mean-square deviation (RMSD) between the redocked poses and the crystallographic ligands was calculated, considering values below 2 Å as indicative of convergence. The search space was defined as a 10 Å sphere centered on the crystallographic ligands within the active site of each protein. The GoldScore function was employed, due to its effectiveness in evaluating varied interactions between ligands and target proteins. The results obtained from molecular docking served as a basis for subsequent molecular dynamic simulations with the aim of evaluating the stability and conformational flexibility of the formed complexes.

#### Molecular Dynamics

To further understand the observed interactions between lamivudine and the molecular targets EGFR, RIPK1 and RIPK3, molecular dynamics simulations were performed. Molecular dynamics allowed us to explore the stability of the complexes over a period of time, providing detailed information on conformational flexibility and binding energy under conditions that mimic the biological environment. This approach was essential to evaluate the behavior of the lamivudine complexes in response to structural fluctuations, identifying specific interactions that support stability or suggest instability at the binding site. Furthermore, we used free energy analysis methods, such as MM/GBSA, to quantify the binding affinity, providing a more accurate estimate of the viability of lamivudine compared to the crystallographic ligands AQ4, Q1A and ZOV.

MD allowed us to observe the conformational adaptations that lamivudine can adopt to maximize its affinity and efficacy. Our results contribute to outline an optimized repositioning strategy, capable of maximizing the therapeutic potential of lamivudine in target systems, thus advancing its development for oncological applications.

The protocol followed methods employed in previous works by our group.^44–47^

The systems were solvated in a cubic water box with a 10 Å buffer, and counterions were added to neutralize the system charges. Additionally, ions were included to reach a saline concentration of 0.15 M, simulating physiological conditions. Each studied system underwent a four-step minimization process: (i) hydrogen atoms; (ii) water molecules and counterions; (iii) hydrogen atoms, water molecules, and counterions; and (iv) the entire system. The steps presented 5,000 iterations using the steepest descent algorithm, followed by an additional 5,000 iterations with the conjugate gradient algorithm, ensuring stability and avoiding unfavorable steric clashes.

After minimization, the systems were slowly heated to 310 K over 800 ps at constant volume, with positional restraints applied to the solute using a force constant of 15 kcal/mol·Å^2^. Density equilibration was performed with a force constant of 10 kcal/mol·Å^2^, followed by an unrestrained equilibration for 500 ps under constant pressure.

During the equilibration phase, the temperature was maintained at 310 K using Langevin dynamics, with a collision frequency of 2 ps^–1^. Pressure regulation was conducted using a Nose-Hoover thermostat and Martyna-Tobias-Klein barostat, chosen to ensure precision in reproducing pressure fluctuations.^48,49^ The SHAKE algorithm^50^ was employed to constrain bond lengths involving hydrogen atoms, while long-range electrostatic interactions were treated using the Particle Mesh Ewald (PME) method.^51^ A 10 Å cutoff was applied for nonbonded interactions.

The production phase consisted of a 100 ns simulation without positional restraints, at a constant temperature of 310 K, with pressure regulated by a Berendsen barostat due to its computational efficiency. Coordinates were saved every 5 ps during the heating and density equilibration phases, and every 50 ps during the equilibration and production phases, for subsequent analyses.

## Results

### Size Determination by Dynamic Light Scattering (DLS)

The DLS results showed the size of PCL nanoparticles loaded with lamivudine (273.6 nm ± 57 nm), as observed in Figure 1. The polydispersity index (PDI) obtained was 0.05.

**Figure 1:**
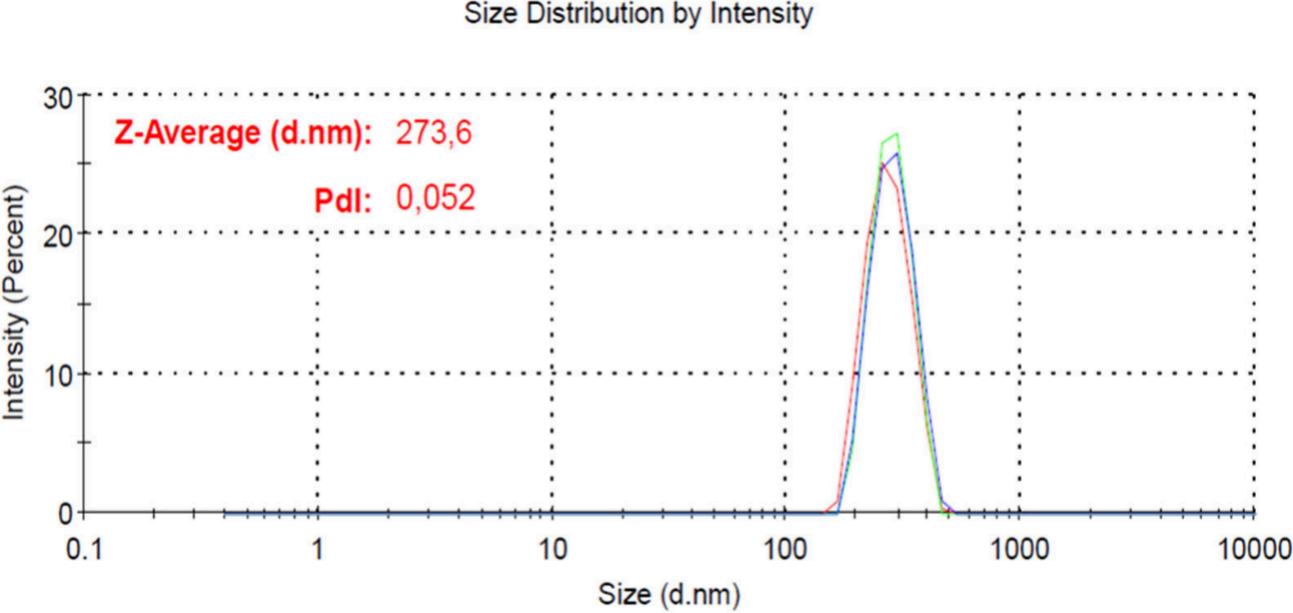
Size by dynamic light scattering. The mean size of PCL nanoparticles for lamivudine nanoparticles. PdI: polydispersity index.

### AFM Ultrastructural Analysis

Figure 2 shows a representative panel of the atomic force microscopy results from the analysis of LamNPs films. Figure 2A shows a height image with a 10-μm scan showing the distribution of the diameters of the nanoparticles obtained in this work. The region marked in the blue dotted square in Figure 2A indicates the area from which the scan in Figure 2B was performed. This 1-μm scan allowed us to assess the texture of an individual nanoparticle. The maps shown in Figures 2C and 2D are three-dimensional representations of the images in Figures 2A and 2B, respectively. The histogram in Figure 2E shows the distribution of nanoparticle diameters. The total number of observed nanoparticles was 62,000, with a mean diameter of 228.8 ± 1.7 nm (mean ± SD).

**Figure 2:**
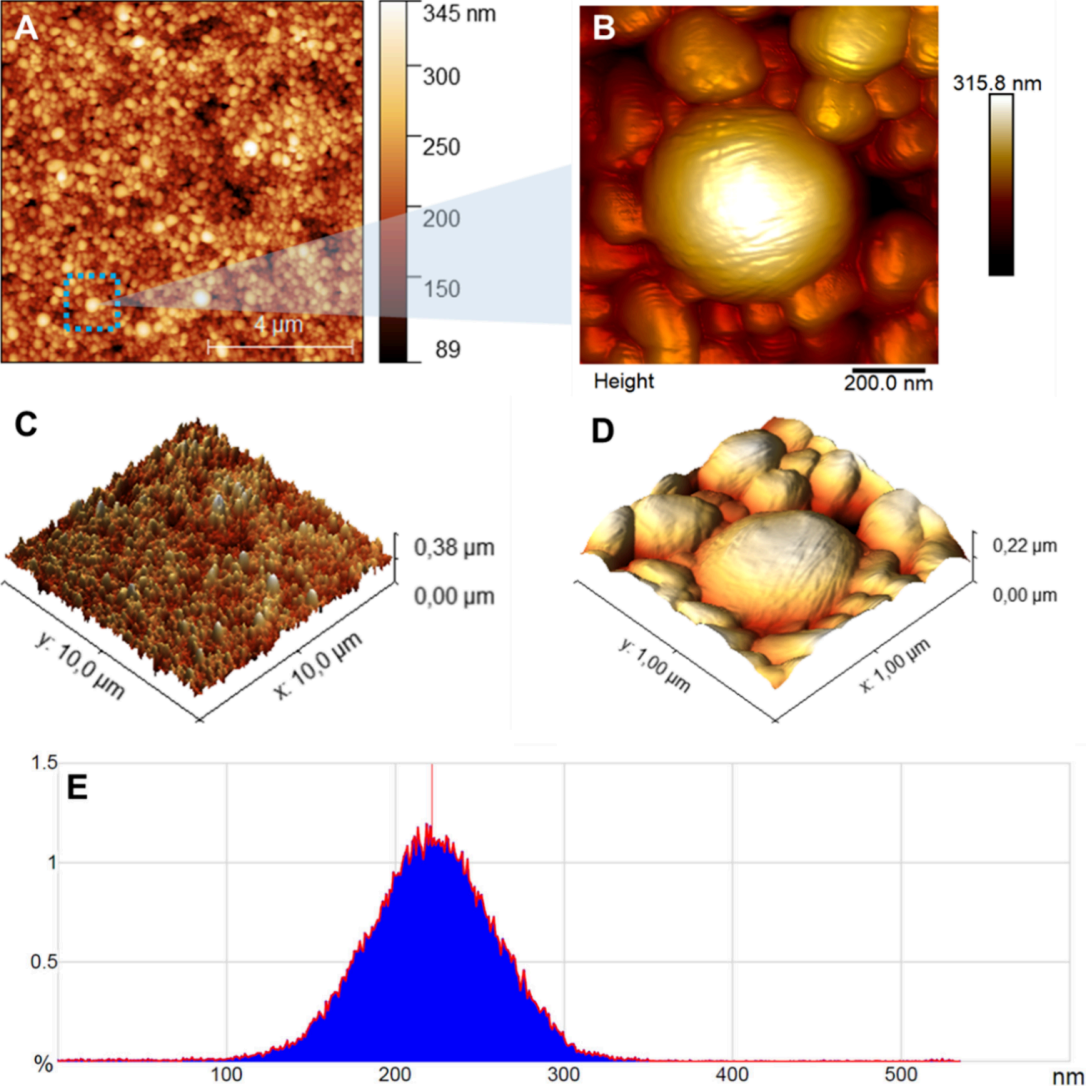
Atomic force microscopy of lamivudine films. A. 10-μm scan showing hundreds of lamivudine nanoparticles—the blue dotted square denotes the region where the height map in Figure 2B was acquired; B. 1-μm scan showing details of nanoparticle surface; C. 3D map related to the image 2A; D. 3D map related to image 2B; E. Histogram of diameter distribution acquired in 10-μm maps. The mean diameter was 228.8 ± 1.7 nm (mean ± SD).

### Encapsulation Efficiency

The encapsulation efficiency of lamivudine in PCL nanoparticles was 89,7 ± 10.3%, which corresponds to 89.7 mg of total lamivudine mass, according to the spectrophotometry technique by which the lamivudine spectrum was measured, where the maximum absorbance peak was identified at 271 nm.

### Release Profile

The release profile test, shown in Figure 3, revealed a rapid initial release of lamivudine from the nanoparticles during the first few hours, followed by a stable release phase with slight declines at 8 and 9 h (Figure 3A). This stability was maintained until the end of the experiment at the 30th hour, indicating a sustained release system. The same figure also shows the pointwise (noncumulative) release, highlighting the release dynamics at specific time intervals. On the other hand, Figure 3B presents the cumulative release profile, emphasizing the progressive increase in the released mass over time, reaching a total of 34.3 ± 0.4 mg of lamivudine, corresponding to 42.3% ± 0.5% of the initial mass. These results demonstrate the effectiveness of the system for the controlled release of the encapsulated compounds, offering therapeutic potential for cancer treatment.

**Figure 3:**
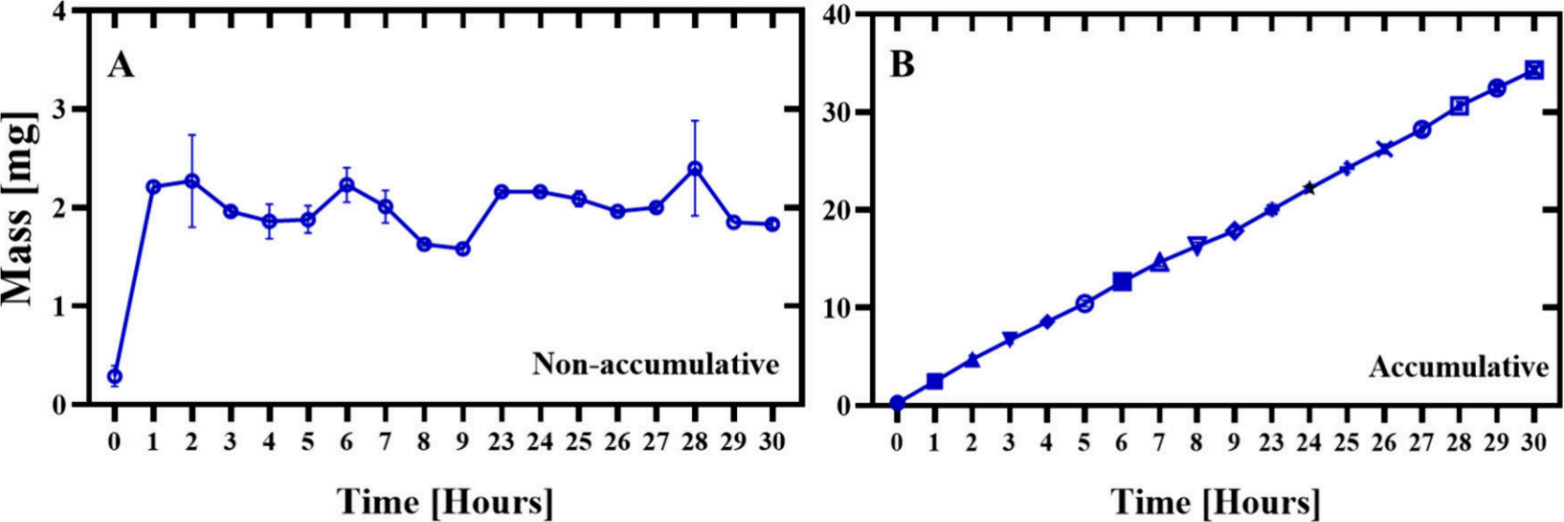
Release profile of lamivudine nanoencapsulated in polycaprolactone. (A) Pointwise (noncumulative) release curve showing a rapid increase in the first hours, followed by a stable release phase until the 30th hour, indicating a sustained release system. (B) Cumulative release profile, highlighting the progressive accumulation of the released mass over time. At the end of the experiment, the total release was 34.3 ± 0.4 mg of lamivudine, corresponding to 42.3% ± 0.5% of the initial mass. The data are expressed as mean ± standard deviation (SD).

### Cell Viability

The MTT assay showed that MDA-MB-231 cells treated with free lamivudine and unloaded PCL nanoparticles at 100 μg/mL did not affect cell viability. However, the use of LamNPs at 100, 50, and 10 μg/mL was able to significantly reduce cell viability by up to 30% when compared to the control (Figure 4).

**Figure 4:**
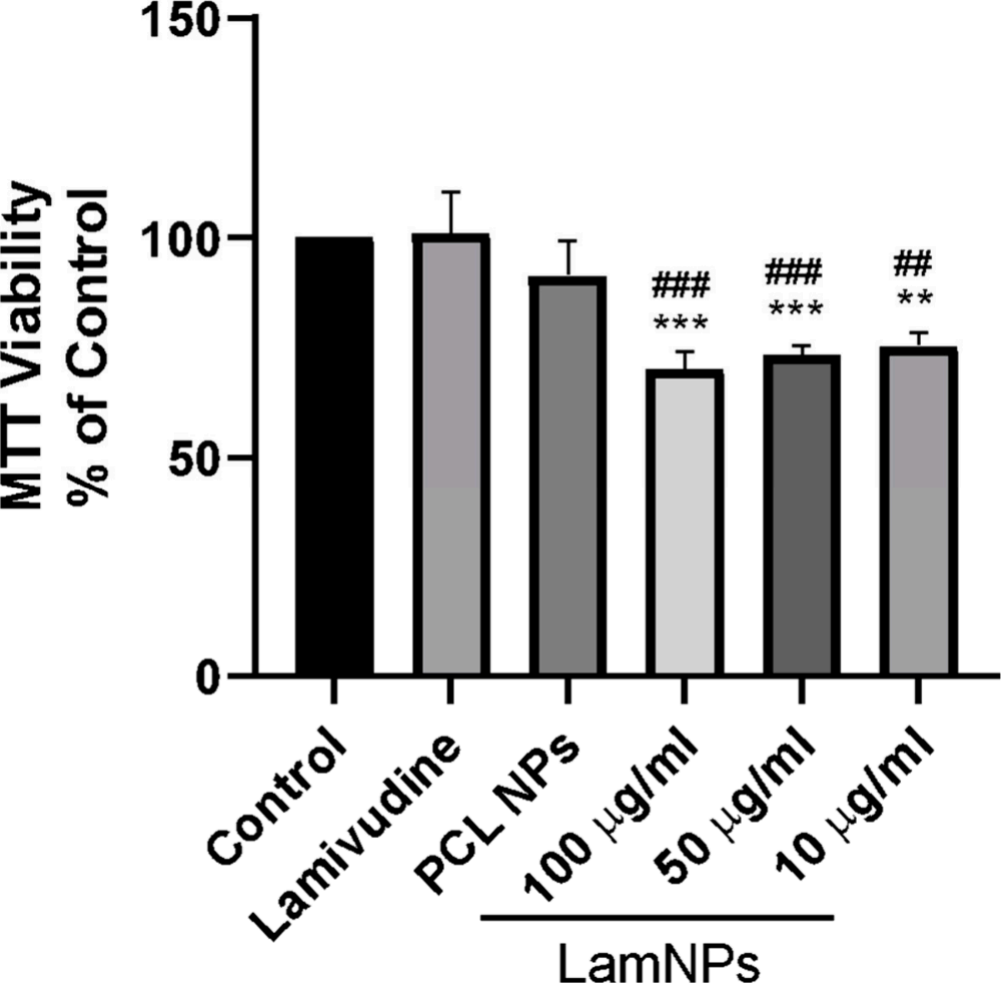
Viability assay (MTT) of MDA-MB-231 lines treated with polycaprolactone (100 μg/mL) and lamivudine NPs at different concentrations (10, 50, and 100 μg/mL) for 24h PCL: polycaprolactone; LamNPs: lamivudine nanoparticles. Data are expressed as the percentage of the control group ± SD (***) *p* < 0.001, (**) *p* = 0.002 (LamNPs vs control); (###) *p* < 0.001, (##) *p* = 0.002 (LamNPs vs free lamivudine).

### *In Vivo* Biodistribution: Radiolabeling with ^99m^Tc and Tissue Deposition

The radiolabeling process was evaluated by radio thin layer chromatography (radio-TLC) for radiolabeling with ^99m^Tc. The results are reported in Table 1.

**Table 1:** Percent Labeling Efficiency over Time after Ascending ^99m^Tc Chromatograms^a^

Time (h)	Labeling (%)
0	99.9 ± 0.002
1	99.9 ± 0.03
2	99.9 ± 0.05
3	99.9 ± 0.05
24	99.0 ± 0.8

a It is possible to observe a high radiolabeling efficacy (>99%) after 24 h.

The biodistribution (Figure 5) showed high uptake in large and small intestines, bladder, left lung and spleen. It is possible to observe small uptake in organs like heart, brain and kidneys.

**Figure 5:**
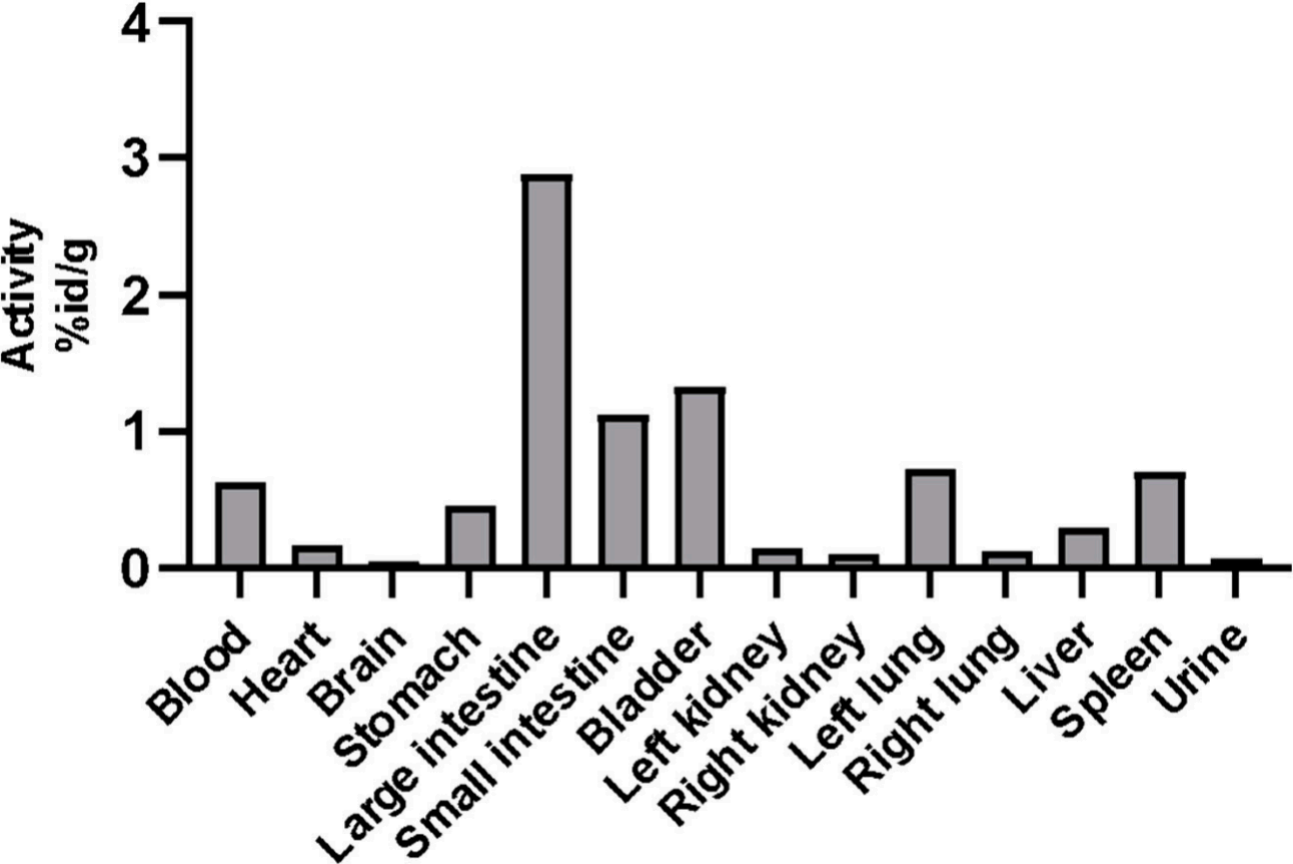
Biodistribution of ^99m^Tc-labeled lamivudine nanoparticles after 24 h, assessed in blood, urine, and various organs, including the heart, brain, stomach, large and small intestines, bladder, left and right kidneys, left and right lungs, liver, and spleen.

### Biochemistry Analysis

The biochemistry results from healthy mice treated with LamNPs are reported in Table 2.

**Table 2:** Results of Biochemical Analysis of Blood Plasma^a^

Parameters (Unit)	Mean ± SD	References
ALT (U/L)	22.4 ± 10.5	46.15 ± 5.62
AST (U/L)	140.1 ± 19.9	121.60 ± 35.93
GGT (U/L)	5.7 ± 1.3	3.40 ± 1.79
CRE (mg/dL)	0 ± 0	0.57 ± 0.006
AMS (U/L)	45.3 ± 18.1	-
LDH-P (mg/L)	1227.8 ± 137.8	-
GLU (mg/dL)	30.8 ± 9.6	147.60 ± 31.76

a Alanine aminotransferase (ALT), aspartate aminotransferase (AST), gamma GT (GGT), creatinine (CRE), amylase (MAS), lactate dehydrogenase pyruvate (LDH-P) and glucose (GLU).^16–18^

### Molecular Docking

The EGFR, RIPK1 and RIPK3 with their respective crystallographic ligands (AQ4, Q1A, ZOV) and lamivudine were studied by molecular docking. Analysis of the binding interactions between the crystallographic ligand AQ4 and lamivudine at the EGFR binding site, as shown in Figure 6 (A and B), suggests potential therapeutic repositioning of lamivudine, a drug already approved for antiviral use and in oncology treatment.

**Figure 6:**
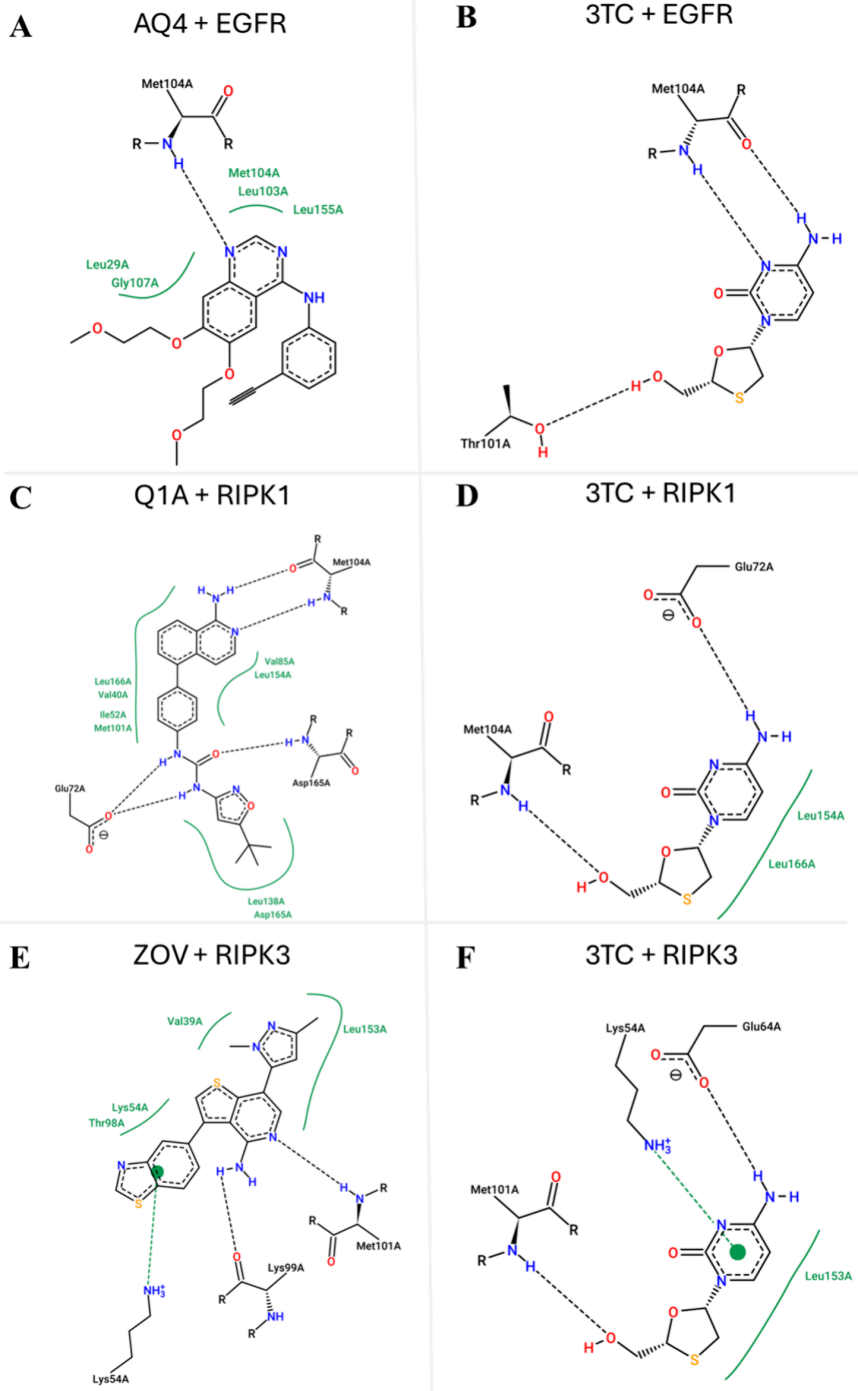
2D diagram of the interactions obtained for the best-ranked docking pose for interaction of crystallographic ligand AQ4 (A) and lamivudine (B) with EGFR; crystallographic ligand Q1A (C) and lamivudine (D) with RIPK1; crystallographic ligand ZOV (E) and lamivudine (F) with RIPK3. The main interactions of the ligands with the active site residues of the proteins are presented, highlighting the hydrogen bonds (dashed black lines) and the hydrophobic contact regions (green curved lines) to observe the critical binding sites involved in the stability of the complexes.

Comparing the interactions of AQ4 and lamivudine (Figure 5 A and B) shows that while lamivudine establishes a hydrogen bond with Met104 and an additional bond with threoninehreonine (Thr) 101, it forms fewer hydrophobic interactions at the EGFR binding site compared to AQ4. Despite this difference, lamivudine has a promising structural complementarity, suggesting that specific adjustments could increase its affinity. Talukder et al. (2025)^52^ demonstrated that structural modifications in promising phytochemicals can optimize hydrophobic interactions and strengthen binding to EGFR.

Lamivudine’s differential interaction with Thr101, a missing interaction in AQ4, may be advantageous in drug resistance scenarios, such as in EGFR mutations that become less responsive to traditional inhibitors. Studies such as that by Yoshizawa et al. (2021)^53^ show that inhibitors with alternative interaction profiles can overcome this resistance by stabilizing distinct target conformations.

Analysis of the binding interactions between the crystallographic ligand AQ4 and lamivudine at the EGFR binding site, as shown in Figure 6 (A and B), highlights the potential of this drug for therapeutic repositioning in oncology.^54^ Originally used in antiviral therapies, lamivudine has a well-established safety profile, which makes its use in new oncology applications attractive, since it reduces development time and cost compared to creating new compounds. This repositioning approach is a common strategy in oncology research, as it allows taking advantage of drugs with a track record of safety and robust clinical data, as exemplified by Ibraheim et al. (2024)^55^ in the context of EGFR inhibitors.

As suggested by Ibraheim et al. (2024), the possibility of lamivudine acting as a dual inhibitor, involving EGFR and VEGFR-2 (Vascular Endothelial Growth Factor Receptor 2),) is interesting, since this synergistic effect could enhance its efficacy by blocking multiple growth and metastasis pathways.^55^ Furthermore, the combined use of lamivudine with other chemotherapeutic agents, as proposed by Shetty et al. (2024),^56^ could reduce toxicity and increase efficacy in cancers resistant to conventional therapies.

Regarding RIPK1, the analysis of the binding interactions between the crystallographic ligand Q1A and lamivudine, shown in Figure 6 (C and D), highlights important aspects of stability and affinity. Q1A establishes multiple hydrogen bonds and hydrophobic interactions, generating strong stability at the binding site and facilitating its inhibitory action. In contrast, lamivudine, despite forming some of the hydrogen bonds observed in Q1A (such as in Methionine104 and Glutamic acid72), presents fewer hydrophobic interactions, which may impact its stability and affinity for RIPK1.

Finally, analysis of the interactions between the crystallographic ligand ZOV and lamivudine at the RIPK3 binding site, depicted in Figure 6 (E and F), reveals the molecular context of RIPK3 in necroptosis and its therapeutic implications. ZOV forms hydrophobic and hydrogen bond interactions, mainly with lysineslysine’s (Lys), such as Lys54, Lys99, and Met101, consolidating its position in the RIPK3 active site. These interactions indicate high affinity and stability, essential for a necrosome complex and fundamental for the induction of necroptosis.

Lamivudine, by forming less robust interactions with hydrophobic residues of RIPK3, has a less stable binding profile than ZOV, suggesting that structural adjustments may improve its ability to promote necroptosis. Thus, although lamivudine presents fewer stabilizing interactions than ZOV, its potential as a RIPK3 modulator can be improved with structural modifications that enhance necroptosis. Incorporation of lamivudine in a nanoencapsulated formulation may maximize its efficacy and distribution to tumor sites, as suggested in necroptosis modulation studies.^57,58^

### Molecular Dynamics

Molecular dynamics (MD) simulations of the complexes formed by EGFR, RIPK1 and RIPK3 with their respective ligands (AQ4, Q1A, ZOV and lamivudine) provided valuable insights into the conformational stability and ligand affinity during the simulation time. The analysis considered parameters such as root-mean-square deviation (RMSD), solvent accessible surface area (SASA), radius of gyration (Rg), root-mean-square fluctuation (RMSF) and binding energies estimated by molecular mechanics/generalized Born surface area (MM/GBSA) (Table 3). These results were correlated with observations from docking simulations, offering an integrated view of the interaction and stability of the ligands with their molecular targets. The interactions observed during docking were compared with the behavior of the complexes during MD, allowing us to assess whether the predicted interactions were maintained or altered.

**Table 3:** Comparative Summary of the Structural Metrics of the EGFR, RIPK1, and RIPK3 Systems^a^

	Root Mean Square Deviation (RMSD)		Solvent Accessible Surface Area (SASA)		Root Mean Square Fluctuation (RMSF)		Radius of Gyration (Rg)	
System	Average (Å)	Standard Deviation (Å)	Average (Å)	Standard Deviation (Å^2^)	Average (Å)	Standard Deviation (Å^2^)	Average (Å)	Standard Deviation (Å^2^)
RIPK1_APO	3.3	0.51	3685.28	110.18	1.28	1.05	20.21	0.14
RIPK1_Q1A	2.64	0.29	3712.32	109.36	2.60	1.10	20.12	0.17
RIPK1_3TCI	2.58	0.34	3844.01	119.53	2.011	1.04	20.39	0.14
EGFR_APO	4.52	0.6	3659.44	166.98	1.48	1.41	20.87	0.38
EGFR_AQ4	6.17	0.65	3676.9	115.22	3.68	1.71	21.1	0.27
EGFR_3TC	6.07	1.4	3698.2	111.92	10.56	4.99	21.36	0.49
RIPK3_APO	2.16	0.28	3520.56	86.4	1.11	0.77	19.3	0.1
RIPK3_ZOV	2.54	0.36	3538.69	104.86	29.56	8.83	19.34	0.1
RIPK3_3TC	1.4	0.23	3652.76	113.89	29.1	8.89	19.89	0.14

a The mean values and standard deviations of RMSD, SASA, RMSF and Rg are presented for each system in its apo forms and complexed with the ligands AQ4, Q1A, ZOV and lamivudine. These structural parameters provide an overview of the conformational stability and surface accessibility of the complexes during MD simulations.

The RMSD plots (Figure 7) highlighted significant differences in the stability of the complexes over the simulation time. In the EGFR system, the complexes with AQ4 and lamivudine presented average RMSDs of 6.17 Å and 6.07 Å, respectively, both higher than the value observed for the APO form (4.52 Å). These results suggest that both ligands cause conformational changes in the receptor, indicating necessary adaptations to accommodate the compounds at the binding site. The behavior of lamivudine, in particular, indicated a certain conformational instability, reflected by its larger RMSD variations over time. Furthermore, the RMSF values underwent greater residual fluctuations in some key regions of the lamivudine-EGFR complex, particularly in the ligand-binding regions and adjacent loops, indicating greater flexibility compared to the complex with AQ4. These fluctuations suggest that lamivudine may not be able to fully stabilize the receptor, which is in line with the observed RMSD values and the lack of stabilizing interactions seen in the docking phase.

**Figure 7:**
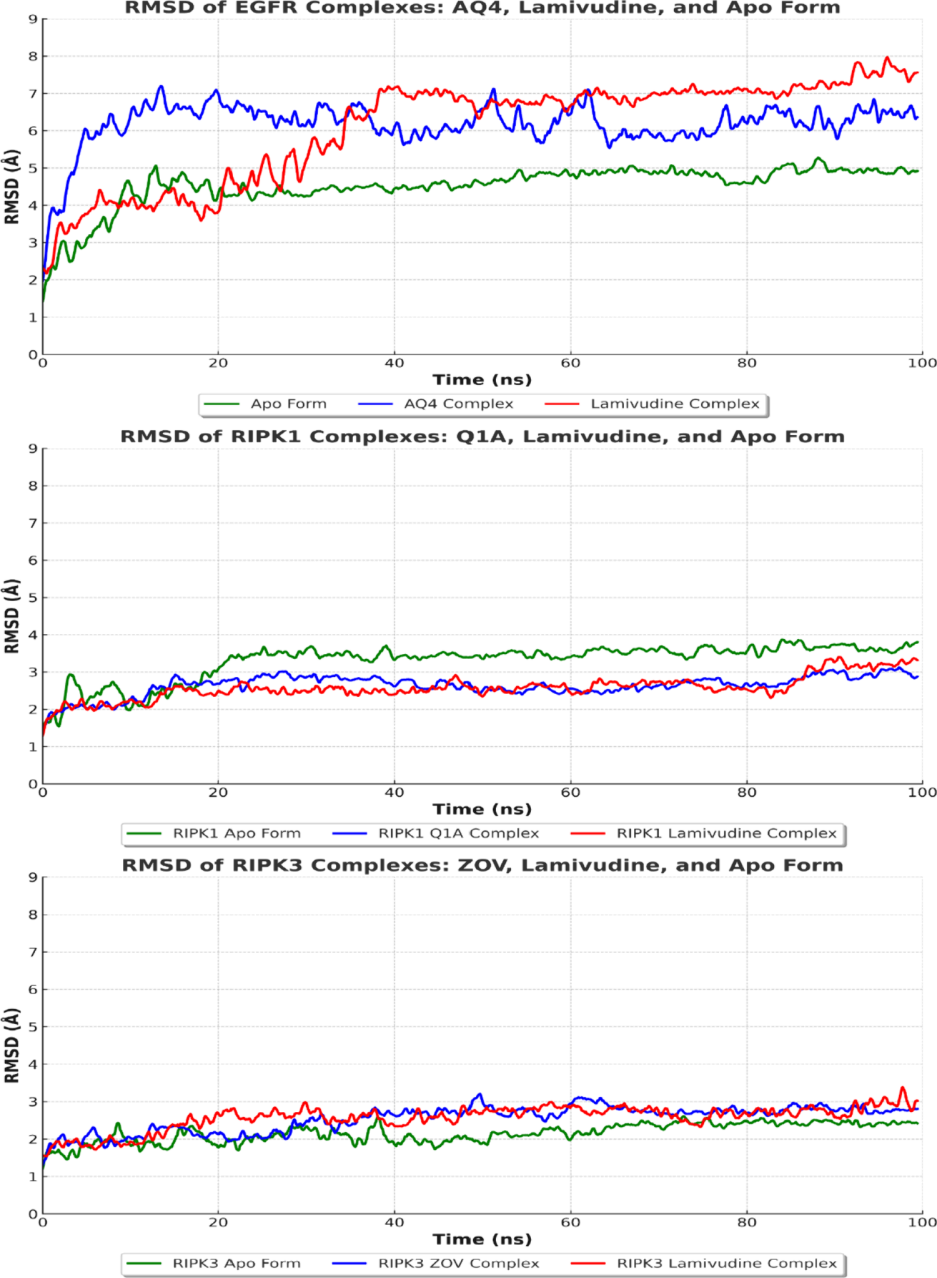
RMSD analysis of the complexes of EGFR, RIPK1 and RIPK3 with their respective ligands AQ4, Q1A, ZOV and lamivudine, as well as the apo forms of each protein, over MD simulation of 100 ns. The green curve represents the apo form, the blue curve denotes the complex with the specific ligand (AQ4 for EGFR, Q1A for RIPK1 and ZOV for RIPK3), and the red curve represents the complex with lamivudine. The RMSD variations over time reflect the conformational stability of the complexes, where larger fluctuations indicate greater flexibility and potential structural instability.

The radius of gyration is a critical parameter to assess the structural compaction of complexes and its relationship with stability.^59^ For complexes with EGFR, the radius of gyration of lamivudine remained slightly higher than that of AQ4 (Table 3), indicating a less compact conformation of the lamivudine-EGFR complex. This lower compaction can be attributed to the lack of additional hydrophobic interactions that could stabilize the complex, as observed in molecular docking (Figures X, A and B). Talukder et al. (2025)^60^ describe how structural modifications in phytochemicals can improve the compaction and stability of complexes, suggesting a viable strategy for lamivudine. The analysis of RMSF values corroborated this observation, showing that critical regions of the lamivudine-EGFR complex presented high fluctuations, suggesting a lower conformational restriction compared to AQ4. Thus, the radius of gyration analysis reinforces the need for structural modifications of lamivudine to improve the stability of the complex.

In the RIPK1 and RIPK3 systems, the gyration radius also showed interesting patterns. For RIPK1, the gyration radius values of the complexes with Q1A and lamivudine were similar, but lamivudine exhibited a slight expansion over time, suggesting less compaction stability. These results are consistent with conformational studies of RIPK1, such as those described by Shetty et al. (2024),^61^ which show that ligands with lower hydrophobic interactions tend to exhibit greater conformational flexibility. This lower compactness is in agreement with the fewer hydrophobic interactions observed from the molecular docking results (Figure 5, C and D), which directly influence the structural organization of the complex. The RMSF values further support this observation, showing larger residual fluctuations in the complex with lamivudine, suggesting less conformational restriction compared to Q1A. In the RIPK3 system, the complex with ZOV maintained a stable and lower gyration radius compared to lamivudine, indicating a more compact and stable structure (Figure 5, E and F). RMSF analysis revealed that lamivudine exhibited larger fluctuations in specific regions of the RIPK3 complex, suggesting that the stability conferred by ZOV results from interactions that limit the flexibility of the receptor.

The SASA analysis of the complexes revealed important information about the solvent exposure of the ligands and its implications for binding affinity. Our molecular dynamics results indicated that free lamivudine exhibited greater solvent exposure compared to standard inhibitors. This finding aligns with our experimental drug release data, whereby the nanoencapsulated system provided protection to the active molecule. For all three systems studied, lamivudine showed higher SASA values compared to the crystallographic ligands, indicating greater solvent exposure and possibly a reduced ability to efficiently occupy the binding sites. This is particularly evident in the EGFR system, where the larger exposure area of lamivudine correlates with a less favorable binding free energy value (ΔG_bind_ = −42.33 ± 7.12 kcal/mol) compared to AQ4 (ΔG_bind_ = −60.11 ± 5.47 kcal/mol), as shown in Table S2 (Supporting Information). This increased exposure was also observed in the RIPK1 and RIPK3 systems, contributing to the lower binding affinity values observed in the MM/GBSA energy and corroborating the docking and RMSF results, which showed lower stability for lamivudine due to the lack of hydrophobic interactions and increased structural flexibility.

The MM/GBSA binding free energy values (Table S2) showed that in all systems the crystallographic ligands presented a higher affinity compared to lamivudine. In the case of RIPK1, Q1A had a binding energy value of ΔGbind = −55.60 ± 6.35 kcal/mol, while lamivudine obtained ΔGbind = −24.40 ± 6.82 kcal/mol, reflecting its lower efficiency in forming stabilizing interactions, such as hydrophobic and hydrogen interactions. The study of Najafov et al. (2018)^62^ suggest that the loss of essential hydrophobic interactions can result in a significant decrease in binding affinity, corroborated by our results. Similarly, in the RIPK3 system, the ΔG value of lamivudine was lower than that of ZOV, highlighting its lower affinity due to the lack of essential hydrophobic interactions observed in docking. The correlation of RMSF values with MM/GBSA also reinforces that the larger residual fluctuations observed in lamivudine negatively impact stability and binding affinity.

The molecular dynamics results provide a clear understanding of the limitation of lamivudine compared to the crystallographic ligands AQ4, Q1A and ZOV, due to the lower number of stabilizing interactions, as evidenced by the high SASA values and larger radii of gyration of the complexes. Lamivudine presented a less compact conformation and higher solvent exposure, which negatively impacts its affinity for the targets.

These factors, together with the MM/GBSA results, suggest that lamivudine requires structural adjustments to improve its affinity and stability. The RMSD and gyration radius data indicate that lamivudine is able to adapt to the binding site, and modifications that favor the formation of more hydrophobic interactions can significantly increase its binding affinity, making it a viable therapeutic option. Despite the limitations, lamivudine demonstrated potential as a candidate for therapeutic repositioning, especially when considering the use of nanoencapsulated formulations.

Several studies in the literature demonstrate that nanoencapsulation has great potential to improve the bioactivity of drugs, by modulating the release and protecting the active molecules, in addition to increasing their bioavailability.^63–65^ By protecting lamivudine from degradation and allowing its gradual release, nanoencapsulation facilitates a more efficient interaction with the active sites of proteins, which is crucial to increase its binding affinity. Modifications that favor more hydrophobic interactions, characteristic of nanoencapsulation, can further enhance the binding affinity of lamivudine to its therapeutic target, making it a more effective option. Although lamivudine has a weaker binding affinity, this does not imply that it is an inferior therapeutic option. Ligands that bind too strongly to the target can lead to bioaccumulation in tissues, resulting in toxicity and adverse reactions. Thus, a moderate binding affinity, such as that observed for lamivudine, may be advantageous since it reduces the risk of cumulative toxicity, providing a safer and more effective therapeutic approach over time.

## Discussion

The data from the dynamic light scattering analysis showed a mean size of 273.6 nm, which was corroborated by the AFM analysis. The polydispersity index (PDI) was equal to 0.05, denoting a small increase in the size of the particles being analyzed, which could be interpreted as monodispersive behavior of the nanoparticles,^66,67^ i.e., the LamNPs, formed by the technique of double emulsion solvent evaporation, are monodispersive. The quality of the formulation can also be seen in the release profile shown in Figure 3. It is possible to observe a 30-h profile with fast release from the beginning to the end of the process. The release of drugs from microparticles and microcapsules depends on parameters such as size, morphology, structure, degree of cross-linking and the characteristics of the *in vitro* environment, including pH, polarity, polymeric interactions and the presence of salts.^68,69^ We observed a release profile that started quickly and remained semilinear until the end of the analyzed period, which suggests use in controlled release systems, as observed in Figure 3A. The cumulative release (Figure 3B) revealed a slow zero-order release profile. Zero-order drug release systems have the potential to overcome the limitations of immediate and first-order release systems, by providing a constant release of the drug. This maintains therapeutic concentrations for a longer period, limiting side effects, reducing dosing frequency and improving patient compliance.^70^

The cytotoxicity assay showed that even at lower concentrations, LamNPs are able to kill breast cancer cells (MDA-MB-231), in contrast to pure lamivudine, which even at higher concentrations showed no effect on the same cells. Although not fully elucidated, the most probable mechanism of action of lamivudine is related to a nonspecific effect on telomerase, which can support its use as an anticancer therapy.^71,72^ The discrepancy between free lamivudine and nanoparticles loaded with lamivudine in terms of cytotoxicity can be explained by the fact that nanoparticles are better internalized into the cells,^73–75^ promoting better action especially due the fact that the transformation of lamivudine into the functional triphosphate form (3TC-TP) occurs in the nucleus and the cytosol.

The biodistribution results showed high uptake in small and large intestines, which can be explained by the administration route. In this case, we believe the high uptake in intestines was due to the passive diffusion during peritoneal injection (mucosa interaction).^76^ It was also possible to observe a high uptake in liver and spleen. This is explained by the fact that nanoparticles are opsonized by proteins that make them more attractive to phagocytic cells, being captured by the monocyte-macrophages and complementary system.^77,78^ This fact corroborates the alterations in the GGT and AST obtained by biochemical analyses. It is important to note, however, that in terms of dosimetry, the high uptake by the intestines, liver and spleen do not pose a problem, since these organs can support high deposits of γ radiation.^79^ Finally, the uptake of nanoparticles in the kidneys and the presence of nanoparticles in urine suggest renal excretion. The creatine value also corroborates that renal excretion was well-supported by the animals.^80^

Regarding the biochemistry analysis, only two parameters were altered with the administration of LamNPs: gamma-glutamyl transferase and aspartate aminotransferase. The former (GGT) is an enzyme found in many organs, including the liver, pancreas, and kidneys, which plays a role in the metabolism of glutathione. It is commonly used as a biomarker for liver disease or liver damage, since elevated levels of GGT in the blood can indicate liver problems.^81,82^ The alteration in the GGT level in the animals was probably due to the brief accumulation of nanoparticles in the mononuclear phagocytotic system (MPS), especially the liver.^83,84^ This event is corroborated by the alteration in aspartate aminotransferase (AST), also known as serum glutamic oxaloacetic transaminase (SGOT), which is an enzyme that is found in high concentrations in liver cells, but is also present in other tissues such as the heart, skeletal muscles, and kidneys.^85,86^ It is involved in the metabolism of amino acids^87^ and is released into the bloodstream when there is liver or muscle cell damage.

The molecular docking and dynamics simulations demonstrated that lamivudine, in its free form, has certain limitations in terms of stability and affinity within the binding sites of critical targets such as RIPK1, EGFR, and RIPK3. This intrinsic instability was particularly evident in its limited hydrophobic interactions and susceptibility to environmental fluctuations, which may compromise its therapeutic efficacy. Although lamivudine showed lower binding affinity than standard inhibitors, its antitumor efficacy arises from (i) the synergy between multiple mechanisms of action; (ii) its optimized delivery via nanoencapsulation, which offsets reduced *in vitro* stability; and (iii) its ability to accumulate intracellularly as a prodrug (3TC-TP), as observed in previous studies ^88^,.^89^ These findings underscore the need for strategies to shield the molecules from adverse conditions and enhance their pharmacodynamic profile.

Nanoencapsulation addresses these challenges by providing a protective environment that stabilizes lamivudine against external factors such as enzymatic degradation and variable pH. The encapsulation not only preserves the structural integrity of the moleculesm, it also optimizes the interactions at the molecular level. For example, while lamivudine forms hydrogen bonds with key residues like Met104 and Glu72 in RIPK1, its lack of sufficient hydrophobic interactions reduces its stability at the binding site. Encapsulation mitigates this limitation by modulating the molecular microenvironment, favoring enhanced retention and improved binding dynamics.

Nanoencapsulation in PCL overcomes two critical limitations of free lamivudine: (i) the rapid renal clearance of hydrophilic drugs,^90^ and (ii) the poor cellular penetration. As demonstrated by Liu et al. (2021),^91^ nanoparticle systems are preferentially internalized by tumor cells via endocytosis, increasing the drug’s intracellular concentration—a mechanism supported by our cytotoxicity results (Figure 4). Furthermore, protection from metabolic degradation^92^ explains the greater stability observed in the release assays (Figure 3).

This dual role of nanoencapsulation as a stabilizing agent and a delivery platform aligns with the study’s purpose of demonstrating how polymeric nanoparticles can amplify the therapeutic potential of lamivudine. The protective barrier provided by the nanoformulation ensures that lamivudine remains functionally active and effectively targeted, even under conditions that would otherwise compromise its performance. These findings reinforce the importance of encapsulation not only to overcome the molecules’ inherent instability, but also to fulfilling its promise as a viable oncological agent.

## Conclusions

This study provides compelling evidence that the administration of lamivudine-loaded polymeric nanoparticles (LamNPs) represents a promising nanotherapeutic strategy for breast cancer treatment, particularly of the triple-negative subtypes. The novelty of our results rests in demonstrating, for the first time, the ability of LamNPs to significantly enhance lamivudine’s anticancer efficacy through improved cellular uptake, controlled release and enhanced interaction with key necroptotic targets—EGFR, RIPK1, and RIPK3. These effects were substantiated by robust *in vitro* cytotoxicity data, favorable biodistribution profiles, and molecular docking and dynamics simulations, together that revealed sufficient structural complementarity to support repurposing lamivudine in oncology.

Importantly, the radiolabeling and *in vivo* biochemistry analyses confirmed the systemic stability and traceability of LamNPs, while also revealing mild hepatobiliary perturbations, reveling the need for further toxicological optimization. The limitations of this study include the use of a single cancer cell line model and the absence of long-term *in vivo* efficacy data. Future investigations should incorporate tumor-bearing models, evaluate chronic toxicity, and explore structure-based modifications to enhance lamivudine’s hydrophobic interactions and target affinity.

Altogether, this study establishes a strong foundation for lamivudine repositioning in oncology via nanoencapsulation, offering a cost-effective path toward expanding the utility of existing antivirals in cancer therapy.

## Supplementary Material



## Data Availability

All data are available by request to the corresponding author.

## Notes

UV–vis spectroscopy data for nanoparticle release profile; MM/GBSA energy calculations for ligand binding to EGFR, RIPK1, and RIPK3 (PDF)
