# Supplementary material for: Exploring the Anticancer Potential of Lamivudine-Loaded Polymeric Nanoparticles: In Vitro Cytotoxicity, Tissue Deposition, Biochemical Impact In Vivo, and Molecular Simulations Analysis
Source: ACS Appl Bio Mater. 2025 May 19;8(6):4815–28. doi: 10.1021/acsabm.5c00182 (PMC12175167; doi:10.1021/acsabm.5c00182)
Supplement: Supplementary file 1 [file mt5c00182_si_001.pdf]

Supporting Information

Exploring the Anticancer Potential of Lamivudine-Loaded Polymeric Nanoparticles: *in vitro* Cytotoxicity, Tissue Deposition, Biochemical Impact *in vivo*, and Molecular Simulations Analysis

Natália Cristina Gomes-da-Silva[1], Alicia de Faria Almeida[1], Patrícia Severino[1], Mohammed Al-Qahtani[2] Luciana Magalhães Rebelo Alencar[3], Pierre Basílio de Almeida Fechine[4], Eduardo Ricci-Junior[5], Laura Fernanda Osmari Vendrame[6], João Augusto Pereira da Rocha[6,7], Solange Binotto Fagan[6], Ralph Santos-Oliveira[1,8*]

1. Brazilian Nuclear Energy Commission, Nuclear Engineering Institute, Laboratory of Nanoradiopharmacy and Synthesis of New Radiopharmaceuticals, Rio de Janeiro, 21941906, RJ, Brazil
2. Cyclotron and Radiopharmaceuticals Department, King Faisal Specialist Hospital and Research Center (KFSHRC), Riyadh,11564, Saudi Arabia
3. Biophysics and Nanosystems Laboratory, Federal University of Maranhão, Department of Physics, São Luis, 65065690, MA, Brazil
4. Grupo de Química de Materiais Avançados (GQMat), Departamento de Química Analítica e Físico-Química, Universidade Federal do Ceará-UFC, Campus do Pici, CP 12100, Fortaleza, 60451-970, CE, Brazil
5. Federal University of Rio de Janeiro, School of Pharmacy, Rio de Janeiro 21941900, RJ, Brazil
6. Franciscan University, Rua dos Andradas, 1614, Santa Maria, 97010-100, RS, Brazil
7. Laboratory of Modeling and Computational Chemistry, Federal Institute of Education, Science and Technology of Pará (IFPA), Campus Bragança, 68600-000, PA, Brazil
8. Rio de Janeiro State University, Laboratory of Radiopharmacy and Nanoradiopharmaceuticals, Rio de Janeiro, 23070200, RJ, Brazil

All correspondence to:

Dr. Ralph Santos-Oliveira

Brazilian Nuclear Energy Commission

Nuclear Engineering Institute

Rio de Janeiro/RJ, Brazil

Rua Hélio de Almeida, 75, Ilha do Fundão-Rio de Janeiro - Brazil

E-mail: roliveira@ien.gov.br

SUPPLEMENTARY MATERIAL

Table S1: Release profile analysis performed by UV-Vis spectroscopy. The results were obtained by analyzing the absorbance at a wavelength of 271 nm. The table expresses the data in triplicate of the masses and their corresponding percentages.

Cumulative							Noncumulative						
**Times (h):**	**Mass**			**%**			**Times (h):**	**Mass/4mL**			**%**		
0	0,23	0,23	0,41	0,28	0,28	0,50	0	0,23	0,23	0,41	0,28	0,28	0,50
1	2,40	2,40	2,67	2,96	2,96	3,29	1	2,18	2,18	2,27	2,68	2,68	2,79
2	5,21	4,43	4,64	6,42	5,45	5,71	2	2,81	2,03	1,97	3,46	2,50	2,42
3	7,15	6,43	6,58	8,80	7,91	8,10	3	1,94	2,00	1,94	2,39	2,46	2,39
4	8,87	8,21	8,63	10,93	10,11	10,63	4	1,73	1,79	2,06	2,13	2,20	2,53
5	10,63	10,06	10,66	13,09	12,39	13,13	5	1,76	1,85	2,03	2,16	2,27	2,50
6	12,93	12,09	13,02	15,92	14,89	16,03	6	2,30	2,03	2,36	2,83	2,50	2,90
7	15,02	14,20	14,83	18,49	17,49	18,27	7	2,09	2,12	1,82	2,57	2,61	2,24
8	16,62	15,84	16,47	20,47	19,51	20,29	8	1,61	1,64	1,64	1,98	2,02	2,02
9	18,20	17,48	17,99	22,41	21,52	22,15	9	1,58	1,64	1,52	1,94	2,02	1,87
23	20,38	19,62	20,14	25,09	24,17	24,80	23	2,18	2,15	2,15	2,68	2,64	2,64
24	22,55	21,77	22,28	27,77	26,81	27,44	24	2,18	2,15	2,15	2,68	2,64	2,64
25	24,73	23,83	24,31	30,46	29,35	29,94	25	2,18	2,06	2,03	2,68	2,53	2,50
26	26,70	25,77	26,28	32,88	31,73	32,36	26	1,97	1,94	1,97	2,42	2,39	2,42
27	28,66	27,76	28,30	35,30	34,19	34,86	27	1,97	2,00	2,03	2,42	2,46	2,50
28	31,08	30,63	30,21	38,28	37,72	37,20	28	2,42	2,87	1,91	2,98	3,53	2,35
29	32,93	32,48	32,06	40,55	40,00	39,48	29	1,85	1,85	1,85	2,27	2,27	2,27
30	34,77	34,26	33,90	42,83	42,20	41,75	30	1,85	1,79	1,85	2,27	2,20	2,27

Table S2: Analysis of the energy differences of the ligands in the EGFR, RIPK1 and RIPK3 systems. The mean values and standard deviations of the energetic contributions, including van der Waals energy (ΔEvdW), electrostaticity (ΔEele), polarization energy (ΔGGB), nonpolar (ΔGnonpol) and total energy estimated by MM/GBSA (ΔGMM/GBSA), are presented. These data reflect the binding affinity of the Q1A, LAMI and ZOV ligands with their targets, providing a detailed view of the energetic interactions that contribute to the stability of the complexes.

Molecule	ΔEvdW	ΔEele	ΔGGB	ΔGnonpol	ΔGMM/GBSA
EGFR_3TC	-21.23	-7.43	17.32	-2.60	-13.95
EGFR_AQ4	-48.01	-21.65	38.46	-6.36	-37.55
RIPK1_3TC	-26.66 ± 2.63	-18.32 ± 5.26	25.19 ± 3.43	-4.59 ± 0.12	-24.39 ± 6.81
RIPK1_Q1A	-58.04 ± 3.13	-39.03 ± 3.77	44.42 ± 2.41	-7.46 ± 0.20	-60.11 ± 5.47
RIPK3_3TC	-21.40 ± 2.51	-46.30 ± 5.36	45.91 ± 3.36	-3.87 ± 0.12	-25.67 ± 6.81

RIPK3_ZOV	-49.54 ± 2.55	-107.17 ±7.76	113.79 ±7.78	-5.56 ± 0.13	-48.49 ± 11.28
